# Low threshold optical bistability at terahertz frequencies with graphene surface plasmons

**DOI:** 10.1038/srep12271

**Published:** 2015-07-21

**Authors:** Xiaoyu Dai, Leyong Jiang, Yuanjiang Xiang

**Affiliations:** 1 SZU-NUS Collaborative Innovation Center for Optoelectronic Science & Technology, Key Laboratory of Optoelectronic Devices and Systems of Ministry of Education and Guangdong Province, College of Optoelectronic Engineering, Shenzhen University, Shenzhen 518060, China

## Abstract

We propose a modified Kretschmann-Raether configuration to realize the low threshold optical bistable devices at the terahertz frequencies. The metal layer is replaced by the dielectric sandwich structure with the insertion of graphene, and this configuration can support TM-polarization surface electromagnetic wave. The surface plasmon resonance is strongly dependent on the Fermi-level of graphene and the thickness of the sandwich structure. It is found that the switching-up and switching-down intensities required to observe the optical bistable behavior are lowered markedly due to the excitation of the graphene surface plasmons, thus making this configuration a prime candidate for experimental investigation at the terahertz range. And the switching threshold value can be further reduced by decreasing the Fermi-level or increasing the thickness of sandwich structure, hence providing a new way for realizing tunable optical bistable devices. Finally, the optical bistability at higher terahertz frequency and the influence of relaxation time under the actual experimental condition on Fermi-level are discussed.

Graphene has outstanding optical and electrical characteristics^1^,^2^,^3^,^4^ and hence shows great potential applications in optoelectronic devices^4^, such as, the optical modulator with the ultrafast modulation speed across a broad range of wavelengths^3^, graphene photodetectors with ultra-broadband and high responsivity at room temperature^5^, graphene touch screens with the improving performance and reducing costs^6^, the high-frequency performance of the epitaxial graphene transistors^7^, the tunable and controllable graphene’s plasmons via gate voltage providing an advantage for over surface plasmons on a metal-dielectric interface^8^,^9^, and the graphene broadband polarizer with an extinction ratio up to ~27 dB in the telecommunications band^10^, etc.

Besides the linear response of the graphene sheet, it also displays excellent nonlinear characteristics. Hendry *et al.* found that the third-order optical susceptibility of graphene is only weakly dependent on the wavelength in the near-infrared frequency range^11^. Gullans *et al.* showed that it was possible to realize significant nonlinear optical interactions at the few photon levels in graphene nanostructures^12^. Sun *et al.* and Bao *et al.* exploited the optoelectronic properties of graphene to realize the saturable absorbers and ultrafast lasers^13^,^14^. Wang *et al.* reported a broad optical limiting (at 532 and 1,064 nm) by liquid-phase exfoliation for nanosecond pulses^15^. Zhang *et al.* showed that graphene was possessed of a giant nonlinear refractive index n_2_ ≃ 10^−7^ cm^2^ W^−1^, almost 9 orders of magnitude larger than bulk dielectrics^16^.

Optical bistability in nonlinear optical systems is a phenomenon exhibited by certain resonant optical structures whereby it is possible to have two stable steady transmission states for the device, depending upon the history of the input^17^,^18^. Such a bistable device has potential in all-optical switching^19^, optical transistor^20^, and optical memory^21^, hence it can be used for high-speed processing of optical signals. A basic issue of the optical bistability is to realize it with threshold as low as possible. Generally, a low threshold requires a larger nonlinear effect.

This larger nonlinear effect can be realized by using the medium with a high Kerr constant or by designing a nanostructure with high local field effect in the nonlinear regime. However, the conventional nonlinear Kerr materials display a very weak nonlinear response. Hence the thick nonlinear media with a large Kerr nonlinear index is generally required in order to achieve large optical nonlinearity. However, the bulk optical bistable device is disadvantage to its application in the integrated optical element. So that optical bistabilities in nanostructures with high local field effect, such as, photonic crystal cavities^22^, sub-wavelength metallic gratings^23^, metallic gap waveguide nano-cavities^24^, negative-index material^25^,^26^, and metamaterial^27^,^28^, have been extensively investigated.

More fortunately, it is shown that graphene has the large nonlinear Kerr index and ultrafast nonlinear response at the broad band of frequencies, which offers the possibility to realize the optical bistable devices under actual experimental conditions. Recently, graphene photonics have been extended to nonlinear optical bistable devices due to its superior third-order nonlinear optical properties. By depositing graphene with silicon photonic crystal cavity, Gu *et al.* achieved an effective nonlinear optical device that could enable ultralow-power resonant optical bistability, self-induced regenerative oscillations and coherent four-wave mixing^29^,^30^. We theoretically investigated the optical bistability of reflection at the interface between graphene and Kerr-type nonlinear substrates^31^. Horvath *et al.* reported observation of optical bistability and enhanced thermal nonlinearity in a graphene–silicon waveguide resonator^32^. Peres *et al.* found that a single layer of graphene showed an optical bistability in the lower THz frequency range for nonlinear graphene suspending in air^33^. Recently, Bao *et al.* demonstrated that graphene nanobubbles offer a new and promising type of optical nonlinear medium to overcome the optical path length limitation of atomically thin two-dimensional films, and so that the optical bistability and all optical switching are obtained in this graphene nanobubbles^34^.

However, the switching threshold value of the optical bistability for nonlinear graphene suspending in air is still high due to the lack of the effective coupling mechanism between the photons and graphene, and it also requires very larger Fermi-level of graphene at the higher THz frequency. These have to be too big of a shortcoming under the actual experimental conditions. Hence in the present paper, we propose a modified Kretschmann-Raether configuration to realize the optical bistable devices with a very low threshold and a low Fermi-level of graphene at the terahertz frequency. In association with the ultrafast optical response and gate-variable optical conductivity, graphene might be expected to open a new possibility of tunable, compact, and low threshold optical bistability as a unique nonlinear optical material.

**The nonlinear response of monolayer graphene**

In the terahertz frequency, the nonlinear optical response of graphene can be described by the exact solution of the relaxation-time Boltzmann equation in a uniform ac electric field^33^. In the limit 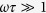, the linear part of the surface conductivity can be expressed as,


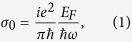


where *ω* is the frequency of the incident light, E_F_ is the Fermi energy, τ is the electron-phonon relaxation time, and ε and 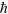 are the universal constants related to the electron charge and the reduced Planck’s constant, respectively. The Fermi energy 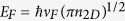 can be electrically controlled by an applied gate voltage due to the strong dependence of the carrier density *n*_*2D*_ on the gate voltage, where *v*_*F*_ *=* 10^6^ m/s is the Fermi velocity of electrons.

In the limit 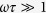 and in the condition of ignoring the third harmonic generation, the third-order nonlinear surface conductivity can be expressed as^33^,


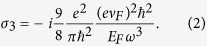


Here, the negative imaginary part of σ_3_ shows the self-focusing type nonlinear response of graphene. Obviously, both σ_0_ and σ_3_ are highly dependent on the work frequency and Fermi energy, which could provide an effective route to achieve an electrically controlled optical bistable phenomenon. Moreover, Peres *et al.* found that the fifth-order nonlinear current occurred in the monolayer graphene, and hence the third-order and fifth-order nonlinear current constituted the nonlinear response of monolayer graphene^33^. In order to simplify the discussion and get better understand of the optical bistability, the fifth-order nonlinearity is not considered in the present paper.

**A modified Kretschmann-Raether configuration for surface plasmons**

Surface plasmons in graphene have been extensively studied both theoretically and experimentally in recent years^8^,^9^. It is well known that the graphene can support p-polarized surface plasmon polariton (SPP) or transverse magnetic (TM) surface wave at the interface of the two dielectrics with the graphene. However, it requires that the graphene should have a positive imaginary part of the surface conductivity. Moreover, it also can support TE-polarized surface electromagnetic wave if the imaginary part of the surface conductivity is negative, however, which is not supporting for an ordinary metal. According to the surface conductivity of graphene in THz frequencies as indicated in Eq. (1), we know that the imaginary part of the linear graphene surface conductivity at the low THz frequency is always positive which leads to the TM surface plasmon wave in the limit 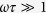, as shown in Fig. 1.

Different from the conventional Kretschmann-Raether configuration, in Fig. 1 we have proposed a modified Kretschmann-Raether configuration where the metal layer has been replaced by the sandwiched structure constituted by the two dielectric slab with the insertion of the graphene sheet. It is clear that this sandwiched structure can support TM-type surface electromagnetic wave. Furthermore, the modified Kretschmann-Raether configuration of attenuated total reflection setup can couple photons into SPPs. And so that the surface plasmon propagates along the dielectric/graphene/dielectric interface. In the present paper, we want to realize the low threshold optical bistability by exciting SPPs. Hence we just discuss the TM-type nonlinear response of the optical bistability.

The presented modified Kretschmann-Raether configuration is shown in Fig. 1, which consists of four different layers of dielectrics. The coupled prism with refractive-index n_p_ and the substrate with refractive-index n_s_ are high refractive index dielectrics, here we assume that n_p_ = n_s_ = 4, and it can be germanium in THz range. The dielectric slabs 1 (n_1_) and 2 (n_2_) with low dielectric constants are inserted between superstrate and substrate. The thicknesses of dielectric slabs 1 and 2 are d_1_ and d_2_, respectively. The graphene sheet is sandwiched between dielectric slabs 1 and 2.

**Theoretical relation of nonlinear optical response**

We choose the *x* axis to be parallel to the interface, and z axis to be perpendicular to the interface. By using the boundary conditions at *z* *=* *−d*_*1*_*, 0*, and *d*_*2*_, and considering the graphene at *z* *=* *0*, we can determine the relation of the incident field and transmitted field,


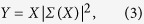


for TM polarization, where *Y* *=* *|H*_*i*_*|*^2^, *X* *=* *|H*_*t*_*|*^2^, and we assume that transmitted magnetic field *H*_*t*_ is purely real. Where


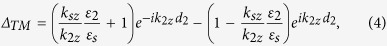



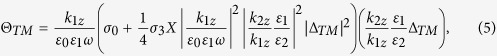



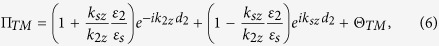



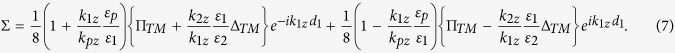


And for TE polarization,


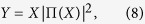


where


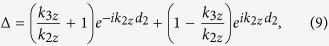



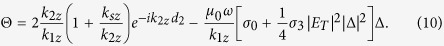



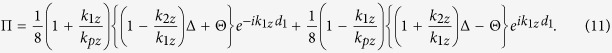


Here we have assumed that transmitted electric field *E*_*t*_ is purely real, and *Y* *=* *|E*_*i*_*|*^2^, *X* *=* |*E*_*t*_|^2^. Where 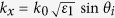, and 
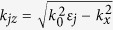
, *j* *=* *p, 1, 2, s. θ*_*i*_ is the incident angle, and *μ*_*0*_ is the magnetic permeability of free space.

Clearly, it follows from Eqs (3) and (8) that incident fields are the multiple value function of the transmitted fields, and hence the optical bistability is appearing at the given appropriate conditions.

**Surface plasmons in the modified Kretschmann-Raether configuration**

The excitation of SPPs with the monolayer graphene in the modified Kretschmann-Raether configuration is demonstrated with the angular reflection and transmission spectra in Fig. 2(a,b), respectively, which are calculated by the modified transfer matrix method^35^ at the very low incident field. Here the incident wave with free space wavelength of *λ* = *300* *um* (*f* *=* *1THz*) is considered, and the dielectrics 1 and 2 are assumed to be same dielectric constants and thicknesses, n_1_ = n_2_ = 1.5, d_1_ = d_2_ = 30 *um*. The Fermi-level is increased from 0.18 *eV* to 0.45 *eV*. It is novel that two resonant dips are clearly resolved in each Fermi-level. The first dip originates from the Brewster angle, which is insensitive to the varying of the Fermi-level of graphene. However, the second reflectance dip at the larger angle is very sensitive to the Fermi-level of graphene, which stems from SPPs of graphene. If the graphene is not considered, the second reflectance dip is disappearing immediately.

It is worthwhile to study the effects of the variations of Fermi-level of graphene on the features of the reflection and transmission spectra. The results, presented in Fig. 2(a,b), respectively, reveal that the change of the Fermi-level of graphene affects the angular and angular width of the reflection and transmission spectra. For E_F_ = 0.18 *eV*, the resonant angle θ_R_ = 78.4°, and full width at half maximum (FWHM) of the resonance Δθ = 0.57°. For E_F_ = 0.45 *eV*, the resonant angle θ_R_ = 34.78°, and FWHM of the resonance Δθ = 5.09°. It is worth noting that for E_F_ = 0.45 eV, the first resonant dip and the second resonant dip start to merge. And for greater Fermi-level, these two resonant dips can form a very wide resonant angular spectrum and they are benefit to the band-pass spatial filtering. To better understand the influence of Fermi-level of graphene on the resonant response, in Fig. 2(c) we give the dependence of the resonant angle and FWHM on the continuously tuning of the Fermi-level of graphene. Clearly, the resonant angles are decreased and FWHM is increased with the increase of the Fermi-level. In the following investigation of the optical bistability, we find that the narrow line width of resonant angle is in favor of the low switching threshold optical bistability.

Besides the dependence of the resonant angular spectra on the Fermi-level of graphene, the resonant angular spectra are strongly influenced by the thicknesses of the dielectric slabs 1 and 2, as shown in Fig. 3(a,b). Here we always assume that d_1_ = d_2_ = d, and E_F_ is fixed at 0.25 eV. We focus on the second resonant dip, and find that the resonant angle shifts to the smaller angle with the increase of thickness d. Meanwhile, the width of resonant angle becomes very narrow which will benefit the low threshold optical bistability. For d = 15 um, the resonant angle θ_R_ = 64.46°, and FWHM of the resonance Δθ = 5.19°; for d = 60 um, the resonant angle θ_R_ = 47.87°, and FWHM of the resonance Δθ = 0.09°. Figure 3(c) displays the resonant angle and FWHM of resonance as the function of the continuously variable thickness d.

**Optical bistability and the influence of initial angle offsets**

In the high incident light field, the nonlinearity in graphene cannot be neglected, hence the surface conductivity of monolayer graphene is written as σ = σ_0_ + Δσ = σ_0_ + σ_3_|E|^2^, where *E* is the electric field amplitude near the graphene, and Δσ = σ_3_|E|^2^ is the change of the surface conductivity induced by the high enough electric field. According to Eq. (2), we have known that the negative imaginary part of *σ*_*3*_ (negative) shows the self-focusing type nonlinear response of graphene. Hence the surface conductivity is always decreased with the increase of the incident electric field. It’s very important that the angular position of SPPs is related to the surface conductivity of graphene, which is shifting toward higher angles as σ is decreased.

The results of the typical calculation for optical bistability are illustrated in Fig. 4, in which we plot transmitted electric field and transmittance versus incident electric field in Fig. 4(a,b), respectively. Here the magnetic field in Eq. (1) has been converted to the electric field according to the relationship of magnetic and electric fields in dielectric, *E*_*i*_ = *η*_*0*_*H*_*i*_*/n*_*p*_, where *η*_*0*_ = 377*Ω* is the impedance in a vacuum. We set the incident angle *θ*_*i*_ = 52°, 54° and 56°, respectively. The resonant angle for E_F_ = 0.25 eV and d = 30 um is *θ*_*R*_ = 50.9°. Hence the initial angle offsets Δ*φ* = 1.1°, 3.1°, and 5.1°, respectively. In the zero incident power the system is in the total internal reflection (TIR) mode for an enough large initial angle offsets, such as θ_*i*_ = 54° and 56°. However, for θ_*i*_ = 52° the initial angle offset Δ*φ* = 1.1° is not large enough to excite the optical bistability. For larger initial angle offset Δ*φ*, it is clear that the hysterical loop occurs. It is indicated that as the input electric field increases, the resonant evanescent electric field in nonlinear graphene increases, and the surface conductivity of graphene decreases resulting in the shifts of the resonant angle to a larger angle *θ*_*R*_. Hence the SPPs mode is approached, and the transmitted electric field increases. When the incident electric field reaches the switching-up threshold, *E*_*up*_ = 5.17 × 10^4^ V/m for *θ*_*i*_ = 54° and *E*_*up*_ = 1.107 × 10^5^ V/m for *θ*_*i*_ = 56°, the transmittance jumps from a very small value to a higher value. This jump leads to the resonant angle *θ*_*R*_ move from below *θ*_*i*_ to slight above *θ*_*i*_. Further increases in electric field move the resonant angle farther from the incident angle *θ*_*i*_ and hence decrease the transmittance. But now as the input electric field is decreased, the resonant angle *θ*_*R*_ decreases and which is gradually approaching to the incident angle and hence gradually approaches to the SPPs condition. When the incident electric field reduces to the switching-down threshold, *E*_*down*_ = 2.42 × 10^4^ V/m for *θ*_*i*_ = 54° and *E*_*down*_ = 2.77 × 10^4^ V/m for *θ*_*i*_ = 56°, the transmittance resonance is realized due to the excitation of SPPs. However, as the input electric field is further decreased, SPPs cannot be maintained and the transmittance point now jumps from the resonant state of SPPs to TIR off resonant state.

To better know about the effect of the initial angle offsets Δ*φ* on the behavior of the optical bistability, we have depicted the curves of the dependence of the switching-up and switching-down optical bistability threshold in Fig. 4(c). It is very significant that the switching-up threshold *E*_*up*_ shifts to a very large incident electric field with the increase of the initial angle offsets Δ*φ*, and nevertheless, the switching-down threshold *E*_*down*_ only has a tiny shift. These results also indicate that the width of the hysterical loop is enlarged obviously.

**Lowered the switching threshold of the optical bistability**

A basic issue of the optical bistability is to realize it with switching threshold as low as possible. The switching-up threshold value for *θ*_*i*_ = 54° is about 1.107 × 10^5^ V/m, so that the intensity |*E*_*i*_|^2^ = 1.225 × 10^10^ V^2^/m^2^, corresponding to 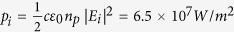; the switching-down threshold value for *θ*_*i*_ = 54° is about *E*_*down*_ = 2.42 × 10^4^ V/m, corresponding to 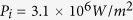. These switching threshold values are much less than the switching threshold values for the nonlinear graphene suspending in free space^33^. And the switching threshold values required in the generation of optical bistability are easy to meet under the actual experimental conditions, such as, Hoffmann *et al.* observed optical birefringence in liquids induced by single-cycle THz pulses with electric field exceeding 100 kV/cm^36^, Hirori *et al.*^37^ obtained maximum THz electric field of 1.2 MV/cm for single-cycle THz pulse, and Beck *et al.*^38^ reported on the generation of impulsive THz radiation with 36 kV/cm vacuum electric field at 250 kHz repetition rate.

Generally, a low switching threshold value requires a large nonlinear effect. This large nonlinear effect can be realized by using the medium with a high Kerr constant or by designing a nanostructure with high local field effect in the nonlinear regime that can enhance the input electric field. According to Eq. (4) and Figs 2 and 3, we have known that the high local field effect in nonlinear graphene can be realized by decreasing FWHM of resonant angle. The sharper the resonant state is, the stronger the local field effect in nonlinear graphene, and the lower optical bistability switching threshold value. The sharp resonant state (i.e. small FWHM) can be realized by decreasing the Fermi-level of graphene or increasing the thickness d, as shown in Figs 2 and 3.

Here, we have chosen two cases as the examples. The results have been illustrated in Fig. 5(a,b). For E_F_ = 0.18 eV and d = 30 um, the resonant angle in the low electric field is *θ*_*R*_ = 78.4° and FWHM of the resonance is Δθ = 0.57°, then we set the incident angle *θ*_*i*_ = *80*°. For E_F_ = 0.25 eV and d = 60 um, the resonant angle in the low electric field is *θ*_*R*_ = 47.8° and FWHM of the resonance is Δθ = 0.09°, then we set the incident angle *θ*_*i*_ = *48.2*°. The switching-up and switching-down threshold values are 4.16 × 10^4^ W/m^2^ and 2.55 × 10^5^ W/m^2^ for E_F_ = 0.18 eV, respectively; and the corresponding switching-up and switching-down threshold values are 2.38 × 10^4^ W/m^2^ and 2.31 × 10^5^ W/m^2^ for E_F_ = 0.25 eV, respectively. These threshold values have been lowered by two orders of magnitude compared to the values in Fig. 4. As a matter of fact, these threshold values can be further reduced by decreasing the Fermi-level and increasing the thickness d at the same time. And that the Fermi-level can be tuned by controlling the bias voltage applied on the graphene sheet, hence which provides a new way to realize the tunable optical bistable devices for future THz optical communication technology.

**Optical bistability at the higher THz frequencies**

In the above discussion, we only consider the hysterical behavior at the low THz frequency. For higher THz frequency, it is difficult to achieve the optical bistability of the monolayer graphene suspending in free space. According to the result in Reference[33], the optical bistability requires that the dimensionless parameter *β* should be larger than 3. Here 
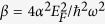
, and α is the fine-structure constant. For larger THz frequency, it needs higher Fermi-level and hence stronger bias voltage applied on the graphene, it may be due to the absence of SPPs in the higher frequencies at the normal incidence. For *f* *=* *2THz*, Fermi-level of graphene E_F_ should be larger than 0.98 eV; for *f* *=* *3THz*, E_F_ should be larger than 1.47 eV. For higher THz frequencies, E_F_ should be even larger. In experimental, it is more difficult to gain the Fermi-level of graphene *E*_*F*_ > 1.0 eV. However, the requirement of the Fermi-level for generation optical bistability can be reduced in our modified Kretschmann-Raether configuration.

We have investigated the angular dependence of the reflectivity for *f* *=* *3THz*, as shown in Fig. 6(a). It is obviously that *E*_*F*_ = 0.53 eV is large enough to excite SPPs, and the angular positions of the reflectance minima shifts to a smaller angle with the increase of the Fermi-level. Figure 6(b) displays the curves of the optical bistability at the different Fermi-level and incident angle. These curves are further evidence of the possibility to realize the optical bistability at higher THz frequencies with low Fermi-level.

**Influence of the relaxation time of carriers on the optical bistability**

Up to now we discuss the optical bistability at the THz frequencies in the limit 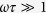, and hence the influence of the relaxation time τ of carriers has been ignored. However in the realistic situations, τ should be included and the linear part of the surface conductivity can be modified as,


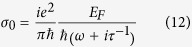


We further assume that the nonlinear response is not influenced by the introduction of the relaxation time τ In Fig. 7(a), we illustrate the dependence of transmittance on the incident angle at the different relaxation time τ of graphene. It is seen clearly that the transmittance is decreased distinctly. Although the relaxation time τ has great influence on the SPPs and hysterical effect, the optical bistability can still exist at the acceptable threshold value of the incident electric field as shown in Fig. 7(b,c). With the introduction of the relaxation time τ, the absorption increases and the transmittance decreases therefore leading to higher energy requirements to excite the optical bistability. Hence as the relaxation time τ decreases, both the switching-up and switching-down threshold values shifts to higher incident electric fields. But these electric fields are still in the range of the actual electric field experimentally as discussed above.

## Conclusions

We have proposed a modified Kretschmann-Raether configuration where the metal layer is replaced by the dielectric sandwich structure with the insertion of monolayer graphene, which can be utilized to realize the tunable optical bistable devices with the ultralow threshold value at the terahertz frequencies due to the excitation of the TM-polarization surface electromagnetic wave. We show that the surface plasmon resonances are strongly dependent on the structure parameters of the geometry and the optical and electrical properties of monolayer graphene, where the resonant angle and the angular width of the resonant state are great influenced by the thickness of the dielectric slabs in sandwich structure and the Fermi-level of the graphene. By setting the enough initial angular offset, the hysterical curves are observed, and the switching-up and switching-down threshold values are controlled by changing the initial angular offset. Moreover, the switching-up and switching-down threshold values can be lowered by decreasing the Fermi-level of graphene or increasing the thickness of the dielectric slabs. Further, we extend our investigation to high THz frequency with appropriate Fermi-level of graphene under the actual experimental conditions, and the influence of the relaxation time of graphene on the hysterical effect is discussed. With giant optical nonlinearities, tunable optical properties, ultra-fast response times, and infinite small thickness permitting the construction of miniaturized devices in integrated optics, graphene optical bistable devices appear to be particularly promising and could potentially open a new possibility of all-optical switching, optical transistor, optical logic, and optical memory.

## Additional Information

**How to cite this article**: Dai, X. *et al.* Low threshold optical bistability at terahertz frequencies with graphene surface plasmons. *Sci. Rep.* **5**, 12271; doi: 10.1038/srep12271 (2015).

## Figures and Tables

**Figure 1:**
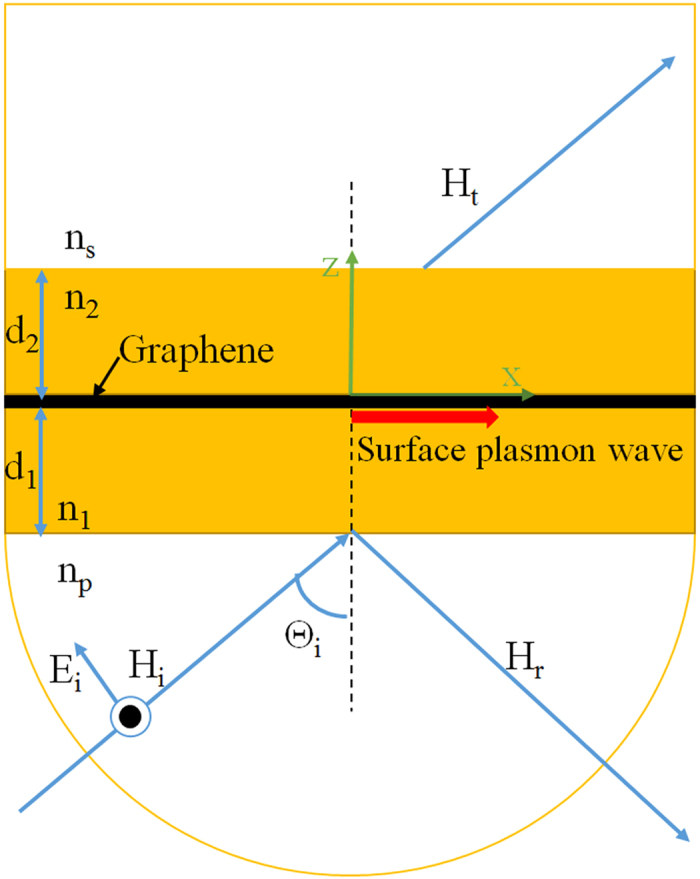
Schematic diagram of a modified Kretschmann-Raether configuration, where the metal layer in Kretschmann-Raether configuration is replaced by the dielectric sandwiched structure with the insertion of graphene sheet. A plane wave of amplitude *H*_*i*_*(E*_*i*_) is incident on the sandwiched structure with incident angle *θ*_*i*_, giving rise to a reflected and a transmitted wave with amplitude *H*_*r*_*(E*_*r*_) and *H*_*t*_*(E*_*t*_), respectively. The surface plasmon wave is excited at the interface of the two dielectric with graphene sheet.

**Figure 2:**
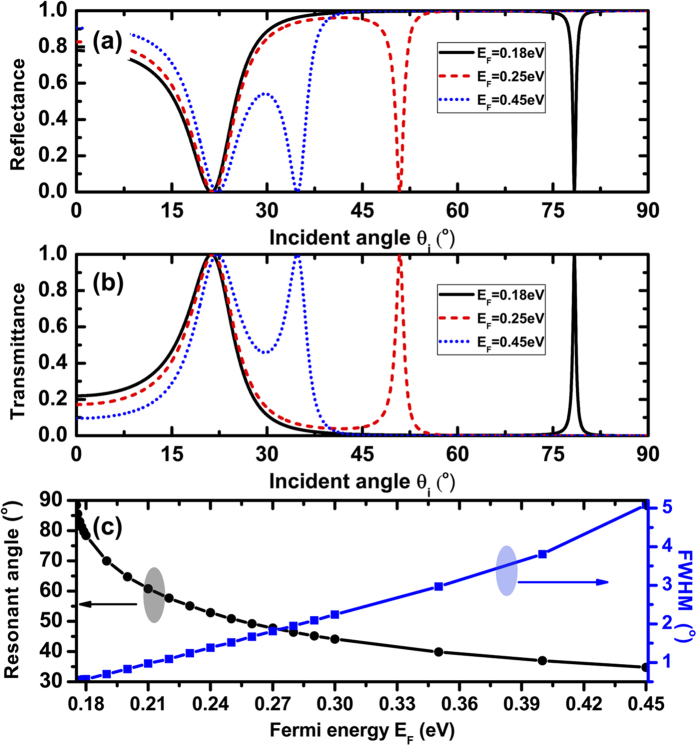
Dependences of the reflectance (**a**) and transmittance (**b**) on the incident angle at the different Fermi-level of graphene; (**c**) Dependences of the resonant angle and FWHM on the Fermi-level of graphene. Where λ=300um, n_p_=n_s_=4, n_1_=n_2_=1.5, d_1_ = d_2_ = 30 um.

**Figure 3:**
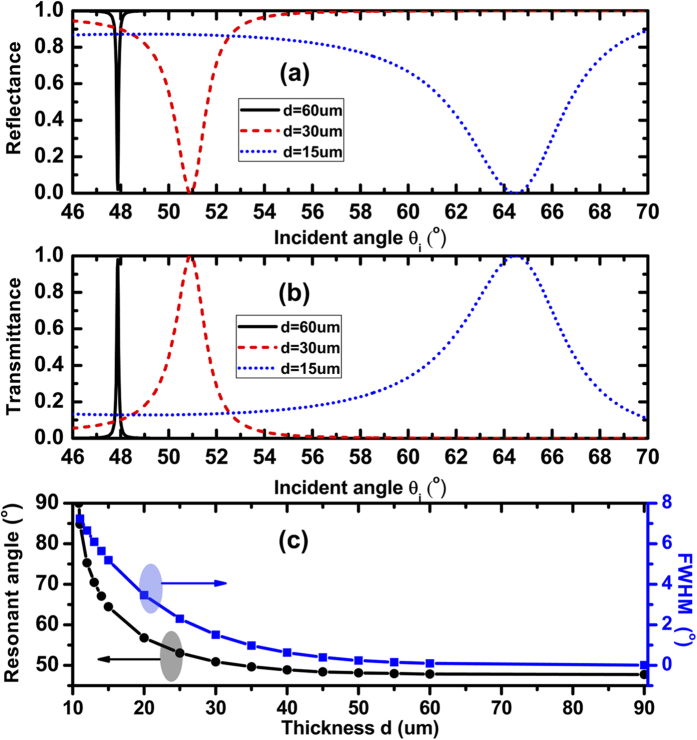
Dependences of the reflectance (**a**) transmittance (**b**) on the incident angle at the different thickness d; (**c**) Dependences of the resonant angle and FWHM of resonant state on the thickness d. Where λ = 300 um, n_p_=n_s_ = 4, n_1_ = n_2_ = 1.5, E_F_ = 0.25 eV.

**Figure 4:**
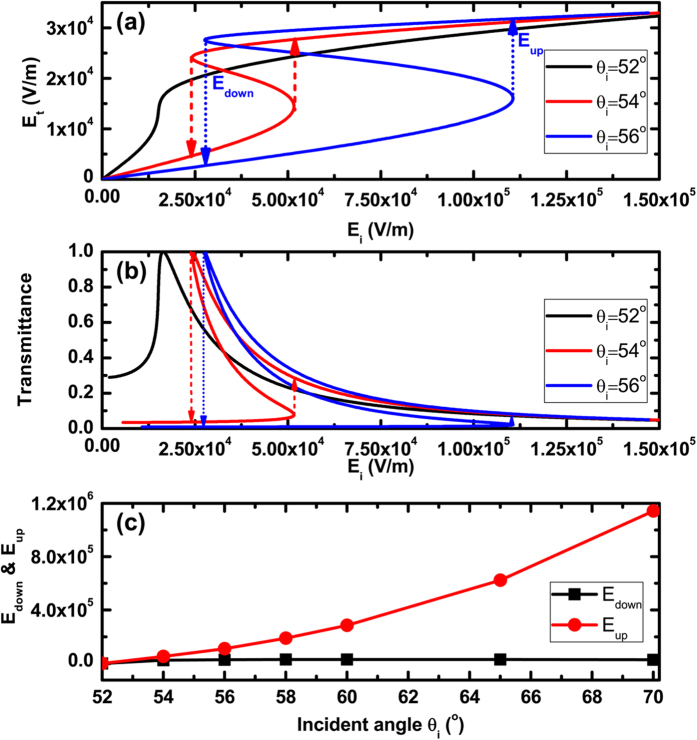
Dependences of transmitted electric field (**a**) and transmittance (**b**) on the incident electric field; (**c**) Influence of the incident angle on the switching-up and switching-down threshold values. Where λ = 300 um, n_p_ = n_s_ = 4, n_1_ = n_2_ = 1.5, E_F_ = 0.25 eV and d = 30 um.

**Figure 5:**
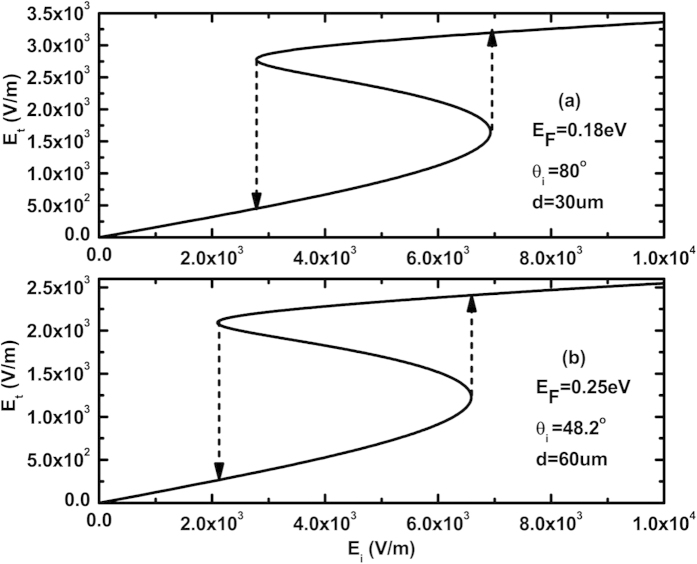
Dependence of transmitted electric field on the incident electric field for E_F_ = 0.18 eV and 0.25 eV, respectively, where λ = 300 um, n_p_ = n_s_ = 4, n_1_ = n_2_ = 1.5. E_F_ = 0.18eV, d = 30 um, θ_i_ = 80° for (**a**) and E_F_ = 0.25 eV, d = 60 um, θ_i_ = 48.2° for (**b**).

**Figure 6:**
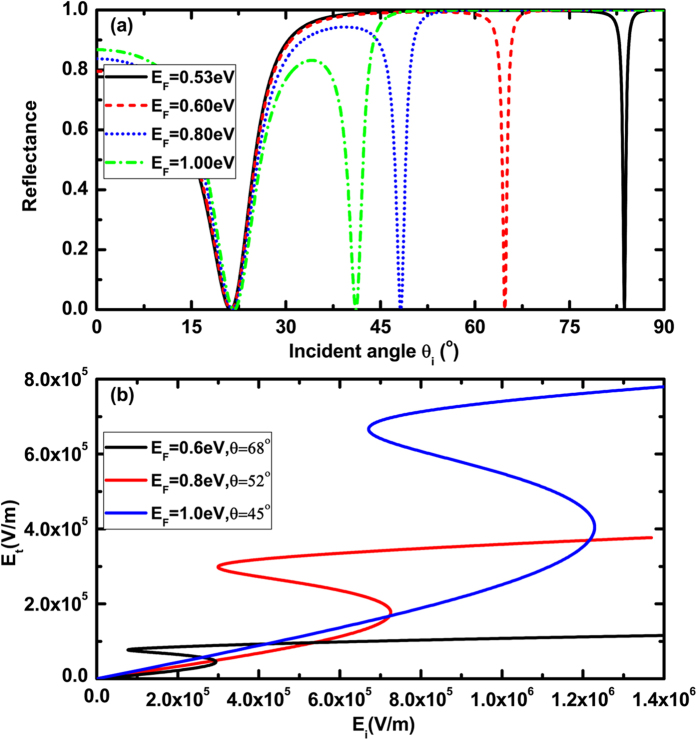
(**a**) Dependence of reflectance on the incident angle at the different Fermi-level of graphene; (**b**) Dependence of transmitted electric field on the incident electric field for E_F_ = 0.6 eV, 0.8 eV and 1.0 eV, respectively, where λ = 100 um, n_p_ = n_s_ = 4, n_1_ = n_2_ = 1.5, d = 10 um.

**Figure 7:**
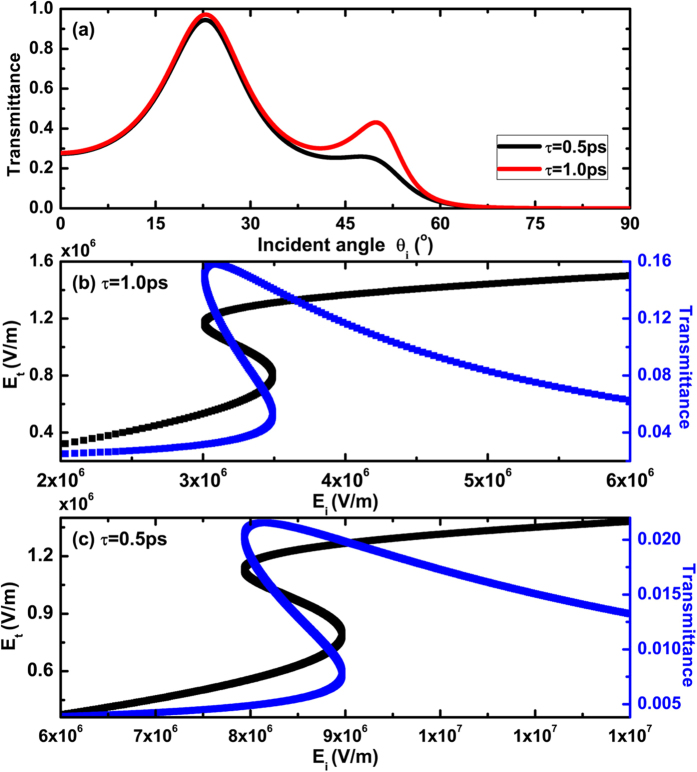
(**a**) Dependence of transmittance on the incident angle at the different relaxation time τ of graphene; Dependences of transmitted electric field and transmittance on the incident electric field in (**b**) for τ = 1 ps and (**c**) for 0.5 ps, respectively. Where λ = 100 um, n_p _= n_s_ = 4, n_1_ = n_2_ = 1.5, d = 5um, E_F_ = 1.0 eV, and θ_i_ = 62° in (**b**), and θ_i_ = 70° in (**c**), respectively.
